# Epigenetic clocks as mediators of health behaviors and mortality in middle-aged and older adults

**DOI:** 10.1016/j.jnha.2025.100602

**Published:** 2025-06-11

**Authors:** Xing-Ling Chen, Qiang-Qiang Zhao, Sheng-Rong Lin, Xing-Ling He, Xiao-Jiao Zhang, Si-Jing Li, Zi-Ru Li, Jia-Hui Chen, Hua Zhang, Xiao-Fang Li, Yue-Hui Zhou, Hui-Li Liao, Shu-Ning Sun, Zhong-Qi Yang, Shi-Hao Ni, Lu Lu

**Affiliations:** a The First Affiliated Hospital, Guangzhou University of Chinese Medicine, Guangzhou 510407, China; b Lingnan Medical Research Center, Guangzhou University of Chinese Medicine, Guangzhou 510407, China; c Guangdong Clinical Research Institute of Chinese Medicine, Guangzhou 510407, China

**Keywords:** Biological aging, Epigenetic age acceleration, Healthy diet, Smoking, Drinking

## Abstract

**Background:**

The impact of healthy lifestyles on epigenetic age acceleration (EAA) and mortality in middle-aged/ senior populations remains unclear. This study investigates associations between lifestyle factors, EAA biomarkers, and mortality risk.

**Method:**

The 2532 adults of 50 years or older that registered in NHANES between 1999–2002.This study evaluated compares first- to third-generation epigenetic clocks (HannumAge, HorvathAge, PhenoAge, GrimAge2, DunedinPoAm) in predicting mortality risk associations between five lifestyle domains (diet, abdominal adiposity, physical activity, smoking, alcohol) and EAA were analyzed via multivariable regression, with mediation models testing EAA’s role in lifestyle-mortality relationships.

**Results:**

Survival curves results identified DunedinPoAm, GrimAge2AA, and PhenoAgeAA as robust biomarkers of accelerated biological aging, independent of chronological age. In multivariable linear regression models, full adherence to healthy behaviors reduced GrimAge2AA by β = −5.55 years, PhenoAgeAA by β = −2.64 years, and DunedinPoAm by β = −0.06 SD, with smoking cessation demonstrating the strongest GrimAge2AA attenuation (10.17 years). Stratified analyses revealed pronounced benefits: cancer patients adhering to healthy diets (β = −0.04 SD, P for interaction = 0.01) and hypertensive individuals reducing smoking (β = −0.05 SD, *P* for interaction = 0.04) showed significant EAA mitigation. The sensitivity analysis is consistent with the original results. Mediation analyses indicated GrimAge2AA accounted for 63.58% of lifestyle-survival associations, DunedinPoAm (44.63%) and PhenoAgeAA (28.45%).

**Conclusions:**

These findings suggest that comprehensive adherence to healthy lifestyle behaviors is associated with reduced epigenetic aging, supporting their potential utility as targets for mortality risk mitigation. And emphasize the utility of epigenetic clocks in precision gerontology.

## 1 Introduction

Long-standing research into the aging process has demonstrated that while social and demographic factors significantly influence disease risk and mortality outcomes, chronological age alone does not capture the complexity of biological aging [1]. This realization has propelled the search for biomarkers that can accurately reflect individual variations in aging speed [[2], [3], [4]] Recent studies increasingly focus on the influence of social determinants on biological aging, exploring how factors like socioeconomic status, lifestyle habits, and environmental exposures intersect with genetic and epigenetic mechanisms [[5], [6], [7], [8]]. A notable advancement in this field is the application of epigenomic analysis, which has enabled the identification of novel indicators of biological age, such as epigenetic clocks [9,10], which derived from DNA methylation (DNAm) patterns at specific CpG sites, act as composite biomarkers that provide molecular insights into aging processes and disease susceptibility [[11], [12], [13]]. Epigenetic clocks have emerged as sensitive tools for assessing biological aging rates, offering a more precise estimate than chronological age alone [14,15].

The refinement of epigenetic clocks has progressed significantly through the use of diverse datasets [12,16,17]. For example, while first-generation clocks such as the Horvath [18,19] and Hannum [20] models accurately estimate chronological age across populations, their predictive power for mortality weakens as their precision in chronological age prediction improves [20,21] This observation suggests that relying solely on chronological age for calibration may not adequately capture the biological elements critical for assessing disease and mortality risks [22]. In response, second-generation clocks like PhenoAge [23]and GrimAge [24] have been calibrated using markers associated with disease and mortality, thus providing a more accurate reflection of biological aging [23,25]. Recent advances include the development of the DunedinPoAm clock [26,27], a third-generation epigenetic measure that utilizes longitudinal assessments of physical and cognitive function, thereby evaluating the rate of biological aging. These clocks reveal substantial variability in biological aging rates among individuals of the same chronological age, with this variability quantified through epigenetic age acceleration (EAA), which represents the difference between epigenetic and chronological age [28]. EAA serves as an indicator of biological aging, highlighting deviations that correlate with disease risk and mortality [8,12].

Epigenetic modifications provide a promising avenue for lifestyle interventions to potentially modulate or even reverse biological aging processes [29,30]. Evidence suggests that lifestyle factors, such as physical activity, dietary habits, body mass index (BMI) management, smoking, and alcohol consumption, significantly influence epigenetic aging [8,31]. Studies have shown that higher BMI and smoking accelerate biological aging [32,33], while positive behaviors like regular exercise and plant-based diets are associated with reductions in epigenetic age [5,34]. The weight-adjusted waist index (WWI) is an anthropometric measure that high WWI values are linked to unfavorable body composition, indicating increased fat mass, and decreased muscle [35] and bone mass [36] that are associated with pathophysiological processes of aging.

Despite these findings, the combined impact of these lifestyle factors has not been comprehensively evaluated in middle-aged and older populations. To address this gap, our study aims to assess the combined effects of multiple healthy lifestyle components, including adherence to a healthy eating index, WWI management, physical activity, smoking status, and alcohol intake, on EAA specifically in individuals aged 50 years and above. This integrated approach fills a critical gap in the literature by providing a holistic evaluation of how lifestyle factors interact to influence biological aging.

Our study utilizes the most recent DNAm data from the National Health and Nutrition Examination Survey (NHANES) in the United States, providing a robust and standardized dataset for analysis. Specifically, our approach involves, (1) evaluating the correlations between five established epigenetic age measures and chronological age to assess the degree of alignment between these metrics; (2) analyzing the associations between lifestyle index scores and epigenetic clock measures to gain insights into how specific lifestyle components influence biological aging; and (3) investigating the mediating role of EAA in the relationship between lifestyle habits and all-cause mortality, with the ultimate goal of advancing our understanding of how modifiable behaviors impact long-term health outcomes and mortality risk in middle to late adulthood.

## 2 Method

### 2.1 Population and study setting

The data for this study were sourced from the NHANES, focusing specifically on data collected between 1999 and 2002. We established a cohort consisting of individuals who, at their initial follow-up, met the following inclusion criteria, (1) were aged 50 years or older, (2) had sufficient data to calculate both chronological age (HannumAge or HorvathAge) and biological age (PhenoAge or GrimAge2) or biological aging rate (DunedinPoAm), and (3) had at least one recorded follow-up for all-cause mortality. Participants were excluded if more than 50% of their lifestyle data (exposure factors) were missing. The first set of data recorded after a participant’s enrollment in NHANES was designated as the baseline. The specific inclusion and exclusion process is further detailed in Supplement eFig. S1.

The study adhered to the revised December 2021 NHANES protocol, which emphasizes the application of sampling weights and design variables in all analyses to account for the survey’s clustered design and differential selection probabilities. To mitigate potential selection bias, the analysis incorporated interview weights for 2-year and 4-year intervals, addressing both the survey's complex design structure and adjustments for nonresponse and post-stratification. These weights allowed for more precise population estimates and adjusted for the oversampling of specific demographic groups. The National Center for Health Statistics Ethics Review Board (NCHS ERB) provided ethical clearance for the use of this data in our analyses.

### 2.2 Exposure

In this study, we evaluated several key lifestyle factors, inducing diet, exercise, smoking, alcohol consumption, and body composition, based on their established associations with health outcomes, including longevity and mortality risk [37]. Adherence levels of each factor were categorized into non-adherence, partial adherence, and full adherence. Information on lifestyle factors was obtained via interviews using questionnaires in the NHANES. Each factor was categorized by adherence levels, which are as follows:

(a) Health dietary adherence, diet was evaluated using the Health Eating Index (HEI) 2020 version, a scoring system designed to assess diet quality according to the alignment with the 2020 Dietary Guidelines for Americans. The HEI includes several components, such as the intake of fruits, vegetables, grains, dairy, and protein foods, as well as moderation of refined grains, sodium, added sugars, and saturated fats. Dietary adherence was divided into tertiles, with non-adherence (0 points) representing the lowest HEI tertile, partial adherence (1 point) for the mid tertile, and full adherence (2 points) for the highest tertile.
(b) Body composition (WWI), was used as a measure of central obesity, calculated by dividing waist circumference by the square root of height (cm/√kg). WWI offers a refined indicator of fat distribution and central adiposity compared to BMI, as it accounts for the proportion of waist size relative to height [36]. Participants were divided into tertiles, with non-adherence (0 points represents ≥11.64 cm/√kg), partial adherence (1 point represents 11.01–11.64 cm/√kg), and full adherence (2 points represents ≤11.01 cm/√kg).
(c) Exercise adherence was measured based on a self-reported questionnaire covering the metabolic equivalent of task for physical activity level (MET-PA), which quantifies the intensity and frequency of physical activity. MET-PA values were divided into tertiles, with non-adherence (0 points represents ≤441 min/week), partial adherence (1 point represents 441−1254 min/week), and full adherence (2 points represents ≥1254 min/week).
(d) Smoking status, participants were divided into three categories, those who had never smoked (scored 2 points for full adherence), those who had smoked more than 100 cigarettes but only smoked occasionally (scored 1 point for partial adherence), and regular smokers (scored 0 points for non-adherence).
(e) Alcohol use, alcohol consumption was also divided into three adherence levels based on daily intake, greater than 20 g/day (scored 0 points for non-adherence), up to 20 g/day (scored 1 point for partial adherence), and no alcohol intake (scored 2 points for full adherence).

The overall healthy lifestyle score was calculated by summing the adherence scores across all lifestyle factors, with a possible total ranging from 0 (non-adherence across all factors) to 10 (full adherence across all factors).

### 2.3 The covariates

The collected covariates included demographic variables and health conditions known to influence aging and mortality. Demographic variables included age (in years), sex (men/women), ethnicity (categorized as non-Hispanic White, non-Hispanic Black, Mexican American, Latin, and other race), marital status (living with a partner/married, never married/separated/widowed/divorced), educational level (college or higher, less than college), and annual household income (categorized as less than $20,000, between $20,000 and $75,000, and more than $75,000). Health conditions included self-reported diagnoses of hypertension, diabetes, cardiovascular disease (CVD), and cancer.

### 2.4 Epigenetic clocks

The primary outcomes of this study involved five distinct types of EAA metrics, calculated as the difference between chronological age and DNAm age estimates, offering comprehensive insights into biological aging. The DNAm ages were derived from methylation patterns at specific CpG sites and included HannumAge, HorvathAge, PhenoAge, GrimAge2, and DunedinPoAm, each capturing unique dimensions of biological aging. The DNAm data for these measures were obtained from blood samples collected during the NHANES 1999–2000 and 2001–2002 cycles.

Blood samples were drawn from NHANES participants aged 50 years and above, with DNA extracted and stored at −80 °C. The extracted DNA was subjected to bisulfite conversion using the Zymo EZ DNA Methylation kit, following Illumina's standard protocols. Converted DNA samples were then hybridized to Illumina Infinium MethylationEPIC BeadChip arrays, which were scanned with the iScan system to produce IDAT files containing methylated and unmethylated signal intensities necessary for DNAm age estimation. The raw IDAT files underwent preprocessing steps, including color correction, background subtraction, and beta mixture quantile (BMIQ) normalization to adjust for probe-type biases and ensure consistent data quality across samples.

For each DNAm clock, distinct bioinformatics pipelines and mathematical models were applied to generate age estimates, with each clock calibrated to emphasize different biological markers and age-sensitive CpG sites. The first-generation clocks were developed primarily to estimate chronological age: HannumAge [20] was derived from methylation patterns in whole blood using a linear regression model based on blood-specific CpG sites, while HorvathAge [19] served as a multi-tissue predictor, leveraging age-sensitive CpGs shared across various tissues to allow broader applicability. We evaluated two second-generation clocks that were trained on disease phenotypes and mortality outcomes: PhenoAge and GrimAge2. PhenoAge [24] incorporates methylation markers linked to morbidity and mortality, thereby reflecting biological health risks beyond simple aging. GrimAge2 [38] is a second-generation mortality predictor calibrated with DNAm surrogates for proteins such as CRP and cystatin C, and also incorporates smoking-related methylation markers. This design offers improved precision in mortality prediction. We also included a third-generation clock, DunedinPoAm [27], which uses a different unit of measurement. DunedinPoAm estimates the pace of biological aging in standard deviation (SD) units based on longitudinal changes in biomarkers of organ system function.

To quantify EAA, HannumAge, HorvathAge, PhenoAge, and GrimAge2 were expressed in units of years as the difference between DNAm-predicted age and chronological age, with positive values indicating accelerated aging and negative values indicating decelerated aging. DunedinPoAm, which directly measures the rate of biological aging rather than epigenetic age, retained its original score. These rate-of-aging values are expressed in SD units representing a faster (positive) or a slower (negative) rate of epigenetic aging (ie, biological aging) relative to the rate of chronological age over time.

### 2.5 Descriptive statistics

Baseline characteristics were presented by age groups categorized as follows, 50 to <58, 58 to <65, 65 to <74, and ≥74 years. Continuous variables were represented as means (with standard deviations), while categorical variables were expressed as numerical values (with corresponding percentages). The multiple imputation method was utilized to handle missing data, enhancing the robustness and reliability of our analyses by reducing bias.

### 2.6 Correlation and regression analyses

Pearson correlations were calculated between 23 type of methylation age (which from National Health and Nutrition Examination Survey 1999–2000 and 2001–2002

DNA Methylation Array and Epigenetic Biomarkers Data Documentation) and chronological age, as well as between EAA and chronological age. The correlation coefficients (r) were reported for each relationship to assess the internal consistency and predictive relevance of each biomarker. Based on these results, five EAA that PhenoAgeAA, GrimAge2AA, DunedinPoAm, HorvathAA, and HannumAA with robust age correlation and significant mortality prediction were selected for subsequent analyses. Propensity score matching was applied to adjust for the effects of age and sex in evaluating the clinical significance of EAA. And evaluate the classification performance by analyzing the area under the curves (AUC) of the five EAA models using time-dependent receiver operating characteristic (ROC) curves. Cox proportional hazards models were then used to estimate the association between five types of methylation age acceleration and all-cause mortality. Hazard ratios (HRs) and 95% CIs were calculated to assess the increased risk of mortality associated with higher EAA, controlling for sex and age.

Multivariable linear regression models were used to assess the association between five lifestyle factors (diet, exercise, smoking, alcohol consumption, and body composition) and overall healthy lifestyle scores with EAA. Lifestyle factors were categorized as non-adherence, partial adherence, and full adherence. Beta values and their corresponding 95% CIs were calculated to represent the change in EAA for each one-unit increase in adherence to lifestyle factors.

### 2.7 Subgroup and sensitivity analyses

To ensure the robustness and validity of our findings, we conducted subgroup analyses across various factors, including sex, age, and comorbid conditions such as hypertension, diabetes, CVD, and cancer. The results were stratified to reveal any potential heterogeneity in biological aging rates among different population subgroups. Using non-adherence as the reference group, we compared the biological aging outcomes of partial and full adherence within each subgroup. Sensitivity analyses were conducted to further assess the stability of our findings, employing the following criteria, (1) only including participants with complete data; (2) excluding participants with extreme lifestyle adherence scores (0 points or 10 points); (3) excluding participants with extreme methylation age accelerations, defined as values below Q1–1.5 IQR or above Q3 + 1.5 IQR; (4) excluding participants with self-reported CVD, cancer, or diabetes; and (5) re-estimating the associations using propensity score matching based on the same covariates applied in the primary models.

### 2.8 Mediation analysis

The mediation analysis was conducted to assess the potential mediating role of EAA in the relationship between lifestyle factors and survival time. This analysis aimed to determine whether the association between lifestyle factors and mortality operates directly or indirectly through changes in DNA methylation. For each lifestyle factor, we evaluated both the direct effect of the factor on survival time and the indirect effect mediated by EAA. The mediation analysis was conducted using bootstrap resampling with 500 simulations to estimate confidence intervals for the direct, indirect, and total effects, which were reported alongside 95% CIs to evaluate statistical significance.

### 2.9 Statistical software

A significance level of a two-sided *P*-value < .05 was considered statistically significant for all tests. All statistical analyses were conducted using R version 3.6.1. Multiple imputation was performed using the "mice" package, Cox regression analyses were conducted using the "survival" package, propensity score matching was performed with the "MatchIt" package, mediation analysis was executed using the "mediation" package, and subgroup analyses utilized the "Publish" package. The "dplyr" and "ggplot2" packages were used for data manipulation and visualization, respectively.

## 3 Results

### 3.1 Correlations between epigenetic clocks and chronological age

**Fig. 1:**
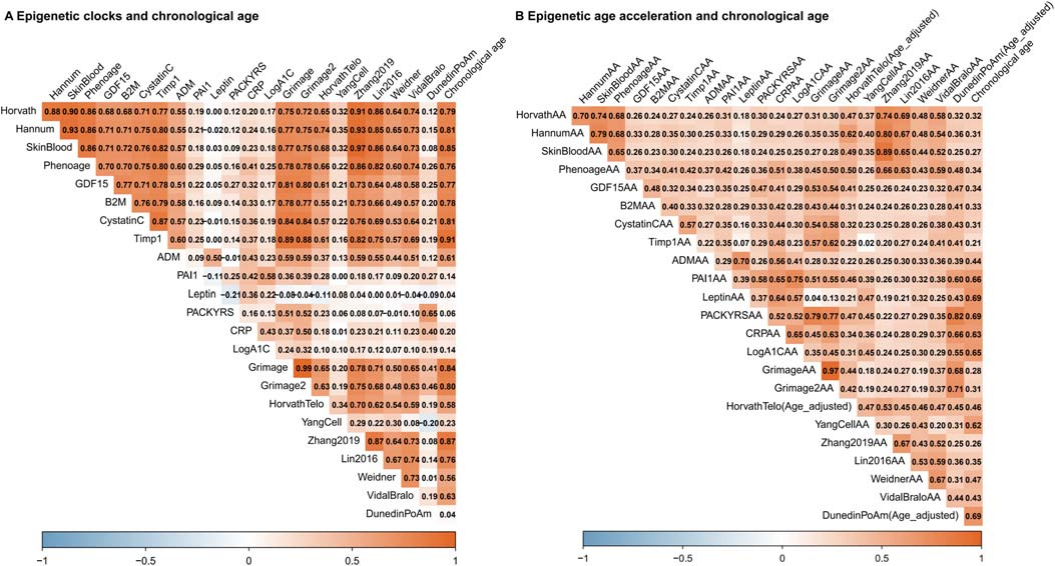
Correlations Between Epigenetic Clocks, Epigenetic Age Acceleration and Chronological Age. Abbreviations: AA, age acceleration.

Pearson correlations were calculated between methylation age and chronological age, as well as between EAA and chronological age across 23 type of methylation age. Our findings revealed that first-generation clocks, such as Horvath age and Hannum age, demonstrated strong correlations with chronological age, affirming their reliability in age prediction. In contrast, newer-generation clocks, particularly PhenoAge, demonstrated weaker correlations with chronological age, possibly reflecting their focus on predicting health outcomes beyond chronological alignment (Fig. 1A). All EAA measures displayed positive associations with minor chronological age differences, suggesting consistent trends across datasets. Notably, the correlation between third-generation measures like DunedinPoAm and chronological age was higher than that of first-generation EAA (Fig. 1B).

### 3.2 Predictive performance of 5 epigenetic age acceleration for mortality risk

We evaluated the clinical relevance of five commonly used epigenetic clocks by assessing their predictive performance for all-cause mortality. The analysis included five EAA measures, HorvathAA and HannumAA (based on chronological age), PhenoAgeAA and GrimAge2AA (based on biological age), and DunedinPoAm, the third-generation methylation clock representing the pace of aging. Time-dependent ROC curves revealed that without age adjustment, DunedinPoAm exhibited the lowest AUC across time points, indicating a limited predictive capacity for mortality relative to the other EAA measures, which consistently maintained AUC values above 0.7. This finding highlights differences in the ability of these clocks to predict mortality over time (eFig. S2).

**Fig. 2:**
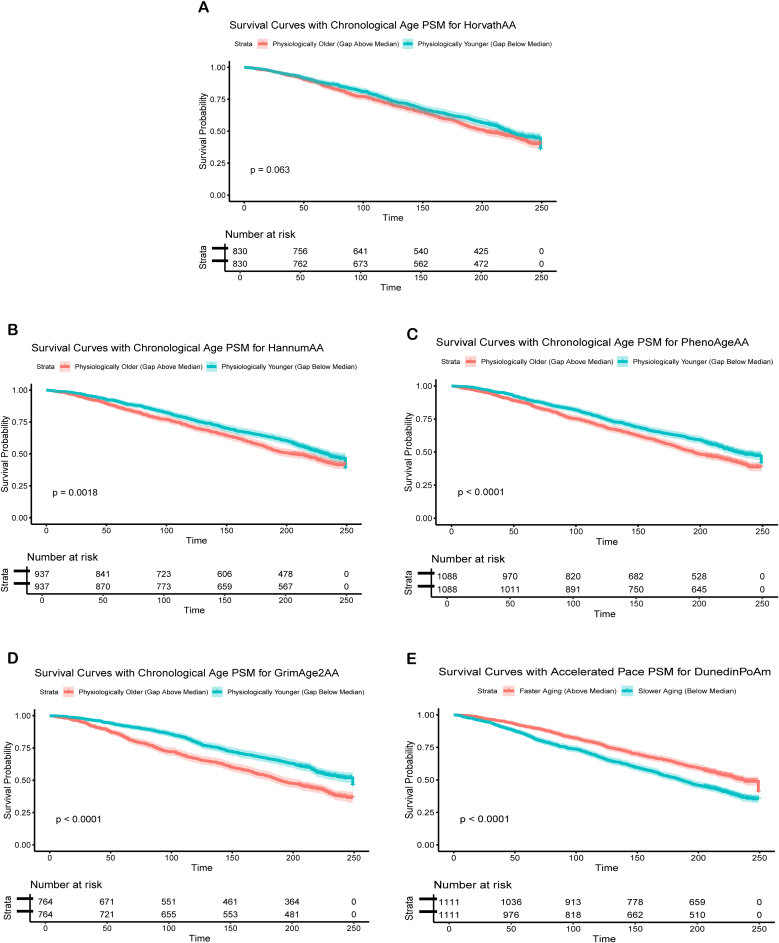
Cox Proportional Hazards Models of 5 Epigenetic Age Acceleration and All-cause Mortality. Abbreviations: AA, age acceleration; PSM, propensity score matching.

Subsequent propensity score matching to adjust for age and sex improved covariate balance, as evidenced by reduced standardized bias in the adjusted analysis [39,40] (eFig. S3). As shown in Fig. 2, GrimAge2AA (HR = 0.64, 95% CI: 0.56–0.74), DunedinPoAm (HR = 0.69, 95% CI: 0.61–0.77), and PhenoAgeAA (HR = 0.78, 95% CI: 0.69–0.87) demonstrated clear risk stratification for all-cause mortality that remained distinct from chronological age. In contrast, although HorvathAA (HR = 0.82, 95% CI: 0.72–0.93) also reached statistical significance, the survival curves closely mirrored those derived from chronological age itself, with minimal separation between risk strata. HannumAA (HR = 0.88, 95% CI: 0.77–1.01) showed a similar pattern and did not achieve statistical significance. These observations indicate that HorvathAA and HannumAA may retain a high degree of dependency on chronological age in mortality prediction. Together, this visual similarity suggests that HorvathAA's predictive signal largely overlaps with that of chronological age, reflecting a potential dependency on age-related variance rather than capturing additional biological risk. GrimAge2AA, DunedinPoAm, and PhenoAgeAA produced more distinct risk stratifications, supporting their utility as age-independent biomarkers of biological aging.

### 3.3 Baseline characteristics of the study population

The baseline characteristics of the study participants (N = 2,532) are summarized by age group (50–58, 58–65, 65–74, and ≥74 years). The mean chronological age increased across the groups, ranging from 53.22 (SD = 2.20) in the youngest group to 79.82 (SD = 3.80) in the oldest. The epigenetic clocks, including HorvathAge, HannumAge, PhenoAge, and GrimAge2, similarly exhibited increasing mean values with age (*P* < .001 for all). DunedinPoAm rates remained relatively stable across age groups (mean = 1.11, *P* = .226). Sex distribution was relatively balanced, with slight variations across groups (*P* = .40). Race/ethnicity showed significant differences, with non-Hispanic White individuals comprising a higher proportion in the oldest age group (57.3%) compared to younger groups (e.g., 38.8% in the 50–58 age group). Conversely, the proportion of Mexican Americans decreased with age (*P* < .001). Lifestyle factors also varied by age. Dietary calorie intake and physical activity levels declined significantly with increasing age (*P <* .001). The mean health eating index improved slightly in older age groups (*P* < .001). Smoking status shifted notably, with the proportion of participants who reported never smoking increasing from 43.9% in the youngest group to 50.4% in the oldest (*P* < .001). Alcohol consumption decreased markedly with age (mean g/d = 11.86 in the 50–58 age group vs. 3.14 in the ≥74 age group, *P* < .001). Educational attainment showed a declining trend, with fewer participants holding a college degree or higher in older groups (*P* < .001). Household income similarly decreased with age, with a higher percentage of participants in the lowest income category (<$24,999) in the oldest group (55.1%) compared to the youngest (32.0%, *P* < .001). Marital status also varied, with a larger proportion of participants being separated in the oldest age group (44.0%, *P* < .001).

In terms of health conditions, the prevalence of diabetes, hypertension, CVD, and cancer increased significantly with age (*P* < .001 for all). For example, CVD prevalence rose from 9.3% in the 50–58 age group to 32.2% in the ≥74 age group. Similarly, cancer prevalence increased from 6.1% to 21.5% across the same age span (Table 1).

**Table 1:** Demographic characteristics and lifestyle of selected epigenetic clock (N = 2532).

	50–58 yr	58–65 yr	65–74 yr	≥74 yr	
Variable	n = 603	n = 591	n = 701	n = 637	*P*-value
**HorvathAge, mean (SD), yr**	57.31(5.38)	63.36(5.16)	69.07(5.79)	76.83(7.83)	<0.001
**HannumAge, mean (SD), yr**	56.80(5.63)	63.26(5.39)	69.35(6.14)	77.88(7.52)	<0.001
**PhenoAge, mean (SD), yr**	44.94(6.78)	51.85(6.51)	58.28(7.70)	67.00(8.86)	<0.001
**GrimAge2, mean (SD), yr**	63.00(5.85)	68.95(5.43)	74.21(5.83)	81.74(5.15)	<0.001
**DunedinPoAm, mean (SD), rate**	1.11 (0.10)	1.11 (0.10)	1.11 (0.09)	1.11 (0.09)	0.226
**Age, mean (SD), yr**	53.22(2.20)	61.37(1.79)	68.82(2.57)	79.82(3.80)	<0.001
**Sex, n (%)**					0.400
Male	307(50.9)	303(51.3)	369(52.6)	306(48.0)	
Female	296(49.1)	288(48.7)	332(47.4)	331(52.0)	
**Race/ethnicity, n (%)**					
Mexican American	150(24.9)	214(36.2)	237(33.8)	120(18.8)	<0.001
Non-Hispanic Black	136(22.6)	142(24.0)	153(21.8)	107(16.8)	0.012
Non-Hispanic White	234(38.8)	177(29.9)	251 (35.8)	365(57.3)	<0.001
Latin	53(8.8)	33(5.6)	43(6.1)	34(5.3)	0.054
Others	30 (5.0)	25 (4.2)	17 (2.4)	11(1.7)	0.003
**Dietary calories, mean (SD), kcal/d**	2023.71 (914.35)	1828.9 (816.04)	1725.87 (735.07)	1593.32 (628.74)	<0.001
**Health eating index,mean (SD)**	51.60 (12.98)	52.38 (12.73)	55.21 (13.04)	55.07 (12.98)	<0.001
**Smoking status, n (%)**					<0.001
Almost Everyday	130(21.6)	98 (16.6)	81 (11.6)	27(4.2)	
Sometimes	208 (34.5)	238 (40.3)	290 (41.4)	289 (45.4)	
Never	265 (43.9)	255 (43.1)	330 (47.1)	321 (50.4)	
**Alcohol, mean (SD), g/d**	11.86 (47.88)	7.23 (19.50)	5.82 (20.63)	3.14 (11.01)	<0.001
**Physical Activity, mean (SD), min/wk**	1581.91(2452.16)	1616.72(2494.14)	1301.73(1815.23)	1172.27(1688.98)	<0.001
**WWI, mean (SD), cm/**√kg	11.00 (0.73)	11.27 (0.70)	11.43 (0.73)	11.60(0.75)	<0.001
**Educational attainment, n (%)**					<0.001
College or above	260(43.1)	190(32.1)	216(30.8)	182(28.6)	
High School or Less	343(56.9)	401(67.9)	485(69.2)	452 (71.0)	
No data	0(0.0)	0(0.0)	0 (0.0)	3(0.5)	
**Household income, n (%)**				<0.001	<0.001
Above $75,000	166(27.5)	127 (21.5)	94 (13.4)	71 (11.1)	
$25,000 - $74,999	225 (37.3)	210 (35.5)	239 (34.1)	193 (30.3)	
Under $24,999	193 (32.0)	236 (39.9)	334 (47.6)	351 (55.1)	
No data	19(3.2)	18 (3.0)	34 (4.9)	22 (3.5)	
**Marital status, n (%)**					<0.001
Separated	183 (30.3)	182 (30.8)	232 (33.1)	280 (44.0)	
With Partner	418 (69.3)	407 (68.9)	467 (66.6)	357 (56.0)	
No data	2(0.3)	2 (0.3)	2 (0.3)	0 (0.0)	
**Diabetes, n (%)**					<0.001
Yes	85 (14.1)	136(23.0)	164(23.4)	127(19.9)	
No	518(85.9)	455(77.0)	537(76.6)	510(80.1)	
**Hypertension, n (%)**					<0.001
Yes	217(36.0)	281(47.5)	375(53.5)	347 (54.5)	
No	386(64.0)	310(62.5)	326(46.5)	288(45.5)	
**CVD, n (%)**					<0.001
Yes	56 (9.3)	102(17.3)	145(20.7)	205(32.2)	
No	546(90.5)	489(82.7)	554(79.3)	428(67.8)	
**Cancer, n (%)**					<0.001
Yes	37(6.1)	58(9.8)	111(15.8)	137(21.5)	
No	566(93.9)	533(90.2)	590(84.2)	499(78.5)	

Abbreviations: SD, standard deviation; WWI, weight-adjusted waist index; CVD, cardiovascular disease.

### 3.4 Association between health behaviors and epigenetic age acceleration

**Fig. 3:**
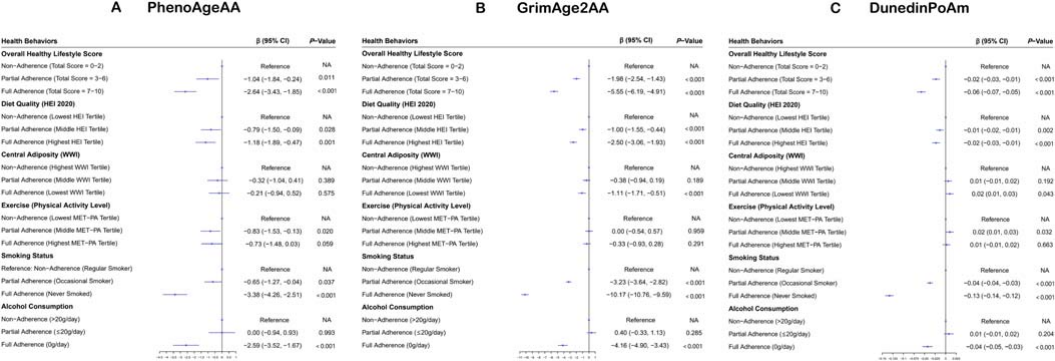
Bivariate Associations Between Healthy Lifestyle and Epigenetic Age in Selected Clocks. Models adjusted for age, sex, ethnicity, marital status, educational level, household income, hypertension, diabetes, cardiovascular disease, and cancer. Abbreviations: AA, age acceleration; CI, confidenceinterval; WWI, weight-adjusted waist index; HEI, health eating index; MET-PA, metabolic equivalent of task for physical activity level.

We evaluated the impact of five key health behaviors (healthy diet, exercise, smoking, alcohol consumption, and maintaining body composition) on biological EAA using PhenoAgeAA, Grimage2AA, and DunedinPoAm as markers (Fig. 3). Full adherence to these healthy lifestyle behaviors was significantly associated with reduced biological EAA across all biomarkers. Specifically, full adherence led to the following reductions, Grimage2AA (β = −5.55 years, 95% CI, −6.19 to −4.91), PhenoAgeAA (β = −2.64 years, 95% CI, −3.43 to −1.85), and DunedinPoAm (β = −0.06 SD, 95% CI, −0.07 to −0.05). Partial adherence also showed notable decreases, Grimage2AA (β = −1.98 years, 95% CI, −2.54 to −1.43), PhenoAgeAA (β = −1.04 years, 95% CI, −1.84 to −0.24), and DunedinPoAm (β = −0.02 SD, 95% CI, −0.03 to −0.01).

**Table 2:** The Mediation analysis of PhenoAgeAA, GrimAge2AA and DunedinPoAm on healthy lifestyle and mortality.

	PhenoAgeAA				GrimAge2AA				DunedinPoAm			
All-cause	Direct Effect	Indirect Effect	Percent, % 95%CI	*P* Value	Direct effect	Indirect effect	Percent, % 95%CI	*P* Value	Direct effect	Indirect effect	Percent, % 95%CI	*P* Value
**Overall Healthy Lifestyle Score**												
Mod vs. Non												
Full vs. Non	−20.64	−94.46	17.93 (7.40–23.72)	<0.001	−102.31	−58.61	63.58 (41.24–80.67)	<0.001	−36.51	−78.11	31.85 (21.75–49.78)	<0.001
**Diet Quality (HEI 2020)**												
Mod vs. Non	−4.86	−46.98	9.37 (-0.26 to 22.81)	0.06	−18.75	−43.32	30.21 (12.51–57.40)	<0.001	−8.33	−45.27	15.55 (5.74–34.67)	<0.001
Full vs. Non	−7.39	−69.77	9.57 (1.58–15.57)	0.01	−45.38	−54.06	45.63 (26.03–59.53)	<0.001	−14.99	−67.20	18.23 (11.30–31.35)	<0.001
**Central Adiposity (WWI)**												
Mod vs. Non	−1.24	22.73	−5.78 (-6.20–58.42)	0.19	−7.54	−24.56	44.34 (21.88–107.44)	<0.001	3.91	20.02	16.35 (4.68–110.06)	0.02
Full vs. Non	2.06	40.64	4.83 (10.30–46.84)	<0.001	−17.19	−36.41	89.41 (34.23–86.00)	<0.001	11.67	34.61	25.21 (14.53–61.71)	<0.001
**Physical Activity Level (MET-PA)**												
Mod vs. Non	−0.36	−10.74	3.24 (-170.30–189.46)	0.52	13.91	−12.18	80.35 (-51.24 to 20.65)	0.95	5.76	−16.51	−53.58 (-458.29–411.23)	0.51
Full vs. Non	−1.49	−18.38	7.49 (-44.13–85.19)	0.20	8.15	−24.73	−49.14 (-9.93–101.27)	0.08	−0.04	−18.49	0.23 (-81.10–109.84)	0.47
**Smoking Status**												
Mod vs. Non	−4.24	−52.30	7.50 (-0.18 to 17.98)	0.06	−59.10	−7.08	89.30 (55.88–169.25)	<0.001	−20.72	−38.78	34.82 (19.84–59.18)	<0.001
Full vs. Non	−14.40	−133.99	9.70 (3.57–11.11)	<0.001	−127.13	−41.68	75.31 (47.81–97.75)	<0.001	−42.29	−99.46	29.83 (15.81–48.48)	<0.001
**Alcohol Consumption**												
Mod vs. Non	0.50	47.18	1.05 (-20.11–19.36)	0.61	14.74	47.63	23.64 (14.37–86.04)	<0.001	8.42	42.69	16.48 (5.28–62.56)	<0.001
Full vs. Non	−11.51	−47.84	19.39 (5.11–34.18)	0.01	−62.97	−13.74	82.09 (22.75–311.00)	0.04	−20.73	−33.22	38.42 (16.74–88.32)	0.01

Note: for the overall healthy lifestyle score, moderate adherence (Total Score 3–6) and full adherence (Total Score 7–10) are compared to non-adherence (Total Score 0–2). For diet quality (HEI 2020), middle and highest tertile adherence are compared to the lowest tertile (non-adherence). For central adiposity (WWI) and physical activity level (MET-PA), middle and lowest tertile adherence are compared to the highest and lowest tertiles, respectively. Smoking status compares occasional smokers and never smokers (full adherence) to regular smokers (non-adherence). Alcohol consumption compares moderate consumption (≤20 g/day) and full abstinence (0 g/day) to high consumption (>20 g/day, non-adherence). The mediation analysis estimates the direct effect of exposure on survival, independent of the mediator (EAA), and the indirect effect mediated through EAA. A GLM modeled the exposure-mediator relationship, while a Weibull survival regression assessed their combined impact on survival. Models adjusted for age, sex, ethnicity, marital status, educational level, household income, hypertension, diabetes, cardiovascular disease, and cancer. Abbreviations: AA, age acceleration; CI, confidence interval; Mod, moderate adherence; WWI, weight-adjusted waist index; HEI, health eating index; MET-PA, metabolic equivalent of task for physical activity level. Non: non-adherence; Mod: partial adherence; Full: full adherence.

Among individual lifestyle factors, smoking cessation had the most profound effect across all EAA measures. Participants who had never smoked exhibited a reduction of up to 10.17 years in biological age as measured by GrimAge2AA (95% CI, −10.76 to −9.59 years), approximately 4 times greater than the reduction associated with a healthy diet (β = −2.50 years, 95% CI, −3.06 to −1.93). Alcohol abstinence (0 g/day) also showed strong associations with reduced biological age acceleration, GrimAge2AA (β = −4.16 years, 95% CI, −4.90 to −3.43), PhenoAgeAA (β = −2.59 years, 95% CI, −3.52 to −1.67), and DunedinPoAm (β = −0.04 SD, 95% CI, −0.05 to −0.03).

Our results also found that adherence to specific behaviors played roles in reducing biological age. For PhenoAgeAA, engaging in weekly moderate exercise contributed to a decrease in biological age (β = −0.83 years, 95% CI, −1.53 to −0.13 years). Grimage2AA was particularly responsive to body composition, with maintaining a healthy weight and reducing abdominal fat associated with a significant reduction in physiological age (β = −1.11 years, 95% CI, −1.71 to −0.51 years). It is appropriate to suggest that the different biological implications reflected by various EAA markers may account for the observed differences in their responses to lifestyle behaviors.

### 3.5 Subgroup and sensitivity analyses of lifestyle adherence and biological aging

Subgroup analyses across age groups, sex, and among participants with diabetes, hypertension, CVD, and cancer provided insights into the heterogeneity of lifestyle adherence effects on DunedinPoAm. Among cancer patients, adherence to a healthy diet was associated with a more pronounced reduction in biological aging rates (β = −0.04 SD, 95% CI, −0.06 to −0.02) than in non-cancer participants (β = −0.02 SD, 95% CI, −0.02 to −0.01, *P* for interaction = 0.01). Similarly, compared to non-hypertensive patients, hypertensive individuals who reduced or ceased smoking still exhibited a significant deceleration in biological aging (β = −0.05 SD, 95% CI, −0.06 to −0.04), highlighting the benefits of smoking cessation within this subgroup (*P* for interaction = 0.04; eFigure S4-S6).

Sensitivity analyses reinforced the robustness of these observations with six models Sequentially exclude participants with incomplete data, extreme lifestyle adherence scores, extreme methylation age acceleration, or those with self-reported CVD, diabetes, or cancer yielded consistent results. And conducted the analysis using different propensity score matching techniques. The findings remained stable across different propensity score matching methods, underscoring the reliability of our analytical approach (eTable S1).

### 3.6 Mediation effects of lifestyle behaviors on survival through biological aging

EAA was found to significantly mediate the association between several lifestyle behaviors and all-cause mortality. The extent of mediation varied across behaviors and DNA methylation clocks, with GrimAge2AA consistently contributing the largest proportion. For the overall healthy lifestyle score, EAA mediated 63.58% of the association with mortality in participants with full adherence (*P* <  0.001), and 59.60% in the moderate adherence group (*P* <  0.001), as measured by GrimAge2AA. The corresponding mediation proportions for DunedinPoAm were 31.85% and 26.58% (*P* <  0.001 for both), and for PhenoAgeAA, 17.93% and 13.52% (*P* <  0.001 for both), respectively.

Across individual lifestyle components, the strongest mediation effects were observed for smoking status, with GrimAge2AA mediating 75.31% of the relationship between full smoking cessation and mortality (*P* < 0.001), followed by DunedinPoAm (29.83%) and PhenoAgeAA (9.70%). When comparing moderate adherence to non-adherence in smoking status, GrimAge2AA mediated 89.30% of the association with all-cause mortality (*P* < 0.001). The high mediation proportions observed for GrimAge2AA are consistent with its design, which includes DNA methylation surrogates for smoking history and inflammation-related proteins, thus increasing its sensitivity to behavior-related mortality pathways. Substantial mediation was also found for alcohol consumption, with GrimAge2AA (82.09%), DunedinPoAm (38.42%), and PhenoAgeAA (19.39%) showing significant effects. For diet quality, EAA mediated 45.63% (GrimAge2AA), 18.23% (DunedinPoAm), and 9.57% (PhenoAgeAA) of the lifestyle-mortality relationship in the full adherence group. Central adiposity (WWI) also showed significant mediation via GrimAge2AA (89.41%) and DunedinPoAm (25.21%). In contrast, physical activity levels exhibited weak and statistically non-significant mediation across all clocks.

Collectively, these findings suggest that a considerable proportion of the beneficial effects of healthy lifestyle behaviors on mortality are explained by slower biological aging, as captured by epigenetic clocks, particularly GrimAge2AA highlighting their potential role as mechanistic biomarkers of lifestyle-related longevity.

## 4 Discussion

This study conducted a comparative evaluation of five EAA—HorvathAA, HannumAge, PhenoAgeAA, GrimAge2AA, and DunedinPoAm—in relation to mortality and modifiable lifestyle factors, using data from NHANES. While all EAA correlated moderately with chronological age, only second- and third-generation EAA (GrimAge2AA, PhenoAgeAA, and DunedinPoAm), which were trained on phenotypic aging and mortality endpoints, demonstrated robust predictive value for mortality risk independent of age. In contrast, first-generation EAA, such as HorvathAA and HannumAA, showed weaker stratification, reflecting their original calibration to chronological age rather than biological aging trajectories [19,20]. Leveraging these refined clocks, we further assessed the impact of five key health behaviors that diet, physical activity, adiposity, smoking, and alcohol use on EAA. Individuals adhering to healthier lifestyles exhibited consistently lower EAA values, particularly in clocks sensitive to functional and morbidity-related changes. Subgroup analyses suggested that dietary adherence was associated with slower aging in cancer patients, and smoking cessation predicted decelerated EAA among individuals with hypertension. Mediation analyses further indicated that EAA partially explained the association between lifestyle behaviors and mortality, especially through GrimAge2AA, highlighting biological aging as a potential intermediary process linking behavior to long-term health outcomes. These findings align with prior studies demonstrating the responsiveness of epigenetic clocks to behavioral exposures [8,27,41] and underscore their utility as surrogate endpoints in future aging and intervention research.

Aging is a multifaceted process characterized by progressive declines in cellular and organ functions, making chronological age an inadequate indicator of underlying biological changes [42]. This has spurred interest in identifying biomarkers that more accurately reflect biological age, such as the epigenetic clock, which estimates biological age by measuring DNA cytosine methylation levels [19]. However, an essential question remains: is aging itself a causative factor for disease? Current theoretical models generally suggest that the passage of time is not a direct cause of disease but rather a contextual framework within which causative factors act [43]. Recent studies support this perspective, indicating that aging clocks can be constructed by quantifying random, stochastic variations that accumulate over time [44]. These findings imply that aging serves as an aggregate measure of accumulated biological and environmental variations, rather than providing direct insight into intrinsic disease mechanisms. Clinically, this suggests that the heightened risk of age-related diseases-such as Alzheimer’s disease, cancer, and cardiovascular disorders-among older individuals arises not from age itself, but from the accumulation of risk factors over time [45]. This understanding positions aging as a cumulative context encompassing various biological and environmental exposures that contribute to disease, rather than a singular causative force. Emerging evidence suggests that lifestyle factors influence DNA methylation via inflammatory, metabolic, and oxidative stress-related pathways. For instance, smoking and poor diet are associated with chronic inflammation and elevated reactive oxygen species, which may induce methylation changes at age-related CpG sites [29]. Physical activity may counteract these effects by modulating immune signaling and enhancing mitochondrial function, thereby promoting a more youthful epigenetic profile [8]. Additionally, dietary components such as polyphenols and folate have been shown to affect methyl donor availability and one-carbon metabolism, directly influencing DNMT activity and methylation patterns [31]. These mechanisms provide plausible biological pathways linking behavior to epigenetic aging.

Our findings highlight strong associations between health behaviors and biological EAA, offering robust evidence that adherence to a healthy lifestyle is associated with lower levels of aging-related biological changes. This protective effect was consistently observed across all biomarkers, highlighting the potential of lifestyle modification as an effective non-pharmacological intervention to mitigate age-associated physiological changes. Notably, our results indicate that smoking cessation was most strongly associated with lower epigenetic age acceleration, aligning with longitudinal research showing that individuals who quit smoking exhibit significantly lower biological EAA over time [46], as assessed through DNA methylation markers. Previous studies have corroborated this connection, demonstrating similar decreases in epigenetic aging indicators among former smokers [47]. Our study extends these findings, revealing that smoking cessation led to a reduction in biological age by up to almost 10 years in GrimAge2AA, substantially more than the impact of other lifestyle behaviors. For example, adherence to a healthy diet was associated with a decrease in biological age of nearly 2.5 years. These results support the importance of smoking cessation in public health strategies based on its strong association with slower biological aging, aimed at mitigating the health impacts of aging and reducing the associated disease burden.

The findings suggest that complete abstinence from alcohol consumption (0 g/day) is associated with the most substantial reductions in biological age acceleration, as demonstrated across all three aging markers. Conversely, moderate alcohol intake appears to correlate with a slight tendency toward accelerated biological aging. This observation aligns with recent research linking alcohol consumption to accelerated biological aging, particularly in middle-aged and older adults [48]. Recent studies have shown that even moderate alcohol consumption has been associated with elevated risks of age-related diseases such as cardiovascular disease, liver dysfunction, and cancers [[49], [50], [51]]. These cumulative aging effects observed in our study underscore the potential benefits of complete abstinence for preserving health and longevity. [52].

Additionally, the findings regarding dietary habits and physical activity also support existing literature on their beneficial effects on biological aging [7,41,53,54], which demonstrated that adherence to health diet patterns (encompasses various components including the intake of fruits, vegetables, whole and refined grains, dairy, and protein foods, while also considering the moderation of sodium, added sugars, and saturated fats) and regular physical activity were associated with decelerated epigenetic aging in middle-aged and older adults. Similarly, regular physical activity, especially moderate to high-intensity exercise, has been consistently associated with slower biological aging by positively influencing metabolic and cardiovascular health. These results further emphasize the role of lifestyle factors that impact physiological systems and, by extension, biological aging.

Studies have underscored the importance of maintaining a healthy body composition, with a specific focus on reducing abdominal fat, as being strongly correlated with lower biological age measured by various epigenetic clocks [24]. Central obesity, beyond overall body weight, has been linked to poor cognitive outcomes and accelerated biological aging [55]. Our findings that Grimage2AA was notably sensitive to central obesity indicators are consistent with these studies. The significant association between a healthier WWI and lower biological age supports the incorporation of body composition measures in lifestyle-based anti-aging strategies.

The subgroup analysis in this study underscores the complex and varied effects of lifestyle adherence on biological aging across different demographic and clinical populations, suggesting that certain subgroups may experience greater benefits from lifestyle changes. Our finding that cancer patients adhering to a healthy diet exhibit a more pronounced reduction in biological aging compared to non-cancer patients aligns with trends observed in meta-analyses [56]. Given the cross-sectional nature of the data, the possibility of reverse causation cannot be excluded that cancer survivors may have adopted healthier dietary practices post-diagnosis, thereby contributing to the observed associations [57]. Similarly, hypertensive individuals who quit smoking showed significant deceleration in biological aging, consistent with research indicating that smoking exacerbates oxidative stress and accelerates vascular aging, especially in those with cardiovascular conditions [58]. While causal relationships cannot be inferred from cross-sectional data, our findings may help identify high-risk subgroups characterized by accelerated biological aging, who could be prioritized for inclusion in future targeted lifestyle intervention trials.

The mediation analysis reveals if lifestyle behaviors impact survival through biological EAA. Full adherence to healthy behaviors significantly mediated the relationship between lifestyle and survival across all three biological age markers, with Grimage2AA showing the strongest mediation effect at 63.58%. This result highlights the marker's sensitivity to body composition and emphasizes the critical role of abdominal obesity and diet in promoting longevity [59]. Meanwhile, DunedinPoAm exhibited strong mediation effects related to smoking cessation, supporting its responsiveness to the reversal of smoking-induced molecular damage, as noted by Belsky et al. [26]. On the other hand, PhenoAgeAA, while showing a lower overall mediation percentage, significantly mediated the effects of alcohol abstinence. This suggests that PhenoAgeAA may be particularly adept at capturing biochemical changes associated with liver health and inflammation reduction, as supported by studies on alcohol-related health risks and systemic inflammation [[60], [61], [62]]. Overall, these differential mediation effects across EAA markers suggest that each marker captures distinct biological pathways influenced by lifestyle behaviors, reinforcing the need for a multidimensional approach to assessing biological age [30]. Such an approach can provide a more comprehensive understanding of how lifestyle interventions influence aging and survival outcomes, improving precision in tailoring interventions for optimal health outcomes [18].

## 5 Strengths and limitations

A key strength of this study lies in its use of a single, nationally representative cohort with high-quality DNA methylation data to concurrently evaluate 23 epigenetic age estimators. This approach enabled a comprehensive and internally consistent comparison of clock performance in relation to both chronological age and mortality risk. Moreover, by examining five clocks across three generations, including those trained on age, phenotypic outcomes, and mortality. We provide novel insights into their relative performance in predicting mortality and their responsiveness to lifestyle factors. The incorporation of mediation analysis further adds depth, offering a novel framework for identifying epigenetic pathways through which lifestyle factors may influence longevity.

Our study has several limitations. First, the cross-sectional design limits our ability to establish a temporal relationship between lifestyle factors and biological aging or infer causality. Longitudinal studies that track life-course patterns of healthy lifestyles and their impact on biological aging are needed to confirm these associations. Second, data collected at a single time point from middle-older adults may reflect a mix of long-term and recently adopted healthy behaviors. Third, the reliance on self-reported measures of MET-PA (physical activities over the previous month) and diet (24 -h dietary recalls) introduces potential recall and social desirability biases, potentially leading to misclassification of lifestyle behaviors. Additionally, the examination of subgroup differences (cancer patients, hypertensive individuals) lacks sufficient sample size information to determine the robustness of these findings. Last, we did not adjust for other factors that may mediate the association of EAA with various clinical outcomes, such as life course psychosocial stressors [63] linked to accelerated epigenetic aging, physiological wear and tear [64], and exposure to environmental toxins [65] that can elicit epigenetic modifications [8]. Future research should aim to address these issues.

## 6 Conclusion

This study demonstrates that modifiable lifestyle behaviors, including smoking cessation, alcohol abstinence, healthy dietary adherence, reduction of central adiposity, and regular physical activity, are significantly associated with lower EAA. By integrating multiple generations of epigenetic clocks, we provide comparative evidence of their responsiveness to behavioral factors and relevance to aging-related risk. Mediation analyses further revealed that EAA partially accounted for the associations between healthy behaviors and all-cause mortality, highlighting its potential role as a biological intermediary linking lifestyle to longevity. While exploratory subgroup findings were observed, the primary contribution of this study lies in advancing the understanding of EAA as a mechanistic marker in lifestyle-aging pathways. These results support the utility of epigenetic biomarkers in future observational and interventional studies aimed at promoting healthy aging.

## CRediT authorship contribution statement

**Xing-Ling Chen and Qiang-Qiang Zhao:** Writing – original draft, Methodology, Formal analysis, Conceptualization. **Sheng-Rong Lin:** Investigation, Data curation. **Xing-Ling He and Xiao-Jiao Zhang:** Investigation, Data curation. **Si-Jing Li, Zi-Ru Li, and Jia-Hui Chen:** Validation, Investigation. **Hua Zhang, Xiao-Fang Li, Yue-Hui Zhou, and Hui-Li Liao** Validation, Investigation. **Shu-Ning Sun, Zhong-Qi Yang, and Shi-Hao Ni:** Supervision, Funding acquisition, Conceptualization. **Lu Lu:** Writing – review & editing, Supervision, Funding acquisition, Conceptualization.

All authors read and approved the ﬁnal version.

## Ethics approval and consent to participate

The National Center for Health Statistics Ethics Review Board (NCHS ERB) provided ethical clearance for the use of this data in our analyses

(https://www.cdc.gov/nchs/nhanes/about/erb.html). All participants provided written informed consent at enrolment.

## Funding sources

This work was supported by a grant from the National Science Foundation of China (No. 82374406), Guangdong Basic and Applied Basic Research Foundation (2023A1515030146), Guangzhou University of Chinese Medicine’s Youth Elite Talents Cultivation “List Unveiling and Leadership”Team Project, 2023 Guangzhou University of Traditional Chinese Medicine First Affiliated Hospital Young and Middle aged Backbone Talent Cultivation Project, The Funding Project from Guangdong Provincial Department of Education (2024KTSCX114).

(https://www.cdc.gov/nchs/nhanes/index.html).

## Data availability

The data are available from the NHANES on request.

## Declaration of competing interest

The authors declare no competing interests.
