# Supplementary material for: Epigenetic clocks as mediators of health behaviors and mortality in middle-aged and older adults
Source: J Nutr Health Aging. 2025 Jun 11;29(7):100602. doi: 10.1016/j.jnha.2025.100602 (PMC12276600; doi:10.1016/j.jnha.2025.100602)
Supplement: Supplementary file 1 [file mmc1.docx]

**Supplementary material**

**eFigure 1**. Flow diagram detailing the inclusion and exclusion criteria for participant selection in the study.

**eFigure 2.** The area under the curves (AUC) of the five epigenetic age acceleration models using time-dependent receiver operating characteristic (ROC) curve without adjusted sex and age.

**eFigure 3.** Propensity score matching was applied to adjust for the effects of age and sex in evaluating the clinical significance of epigenetic age acceleration.

**eFigure 4**. Subgroup analyses for the association between the healthy lifestyle and PhenoAgeAA by sex, age, hypertension, diabetes, cancer and CVD.

**eFigure 5.** Subgroup analyses for the association between the healthy lifestyle and GrimAge2AA by sex, age, hypertension, diabetes, cancer and CVD.

**eFigure 6.** Subgroup analyses for the association between the healthy lifestyle and DunedinPoAm by sex, age, hypertension, diabetes, cancer and CVD.

**eTable 1.** PhenoAgeAA，GrimAge2AA and DunedinPoAm sensitivity analysis Model 1-6.

**
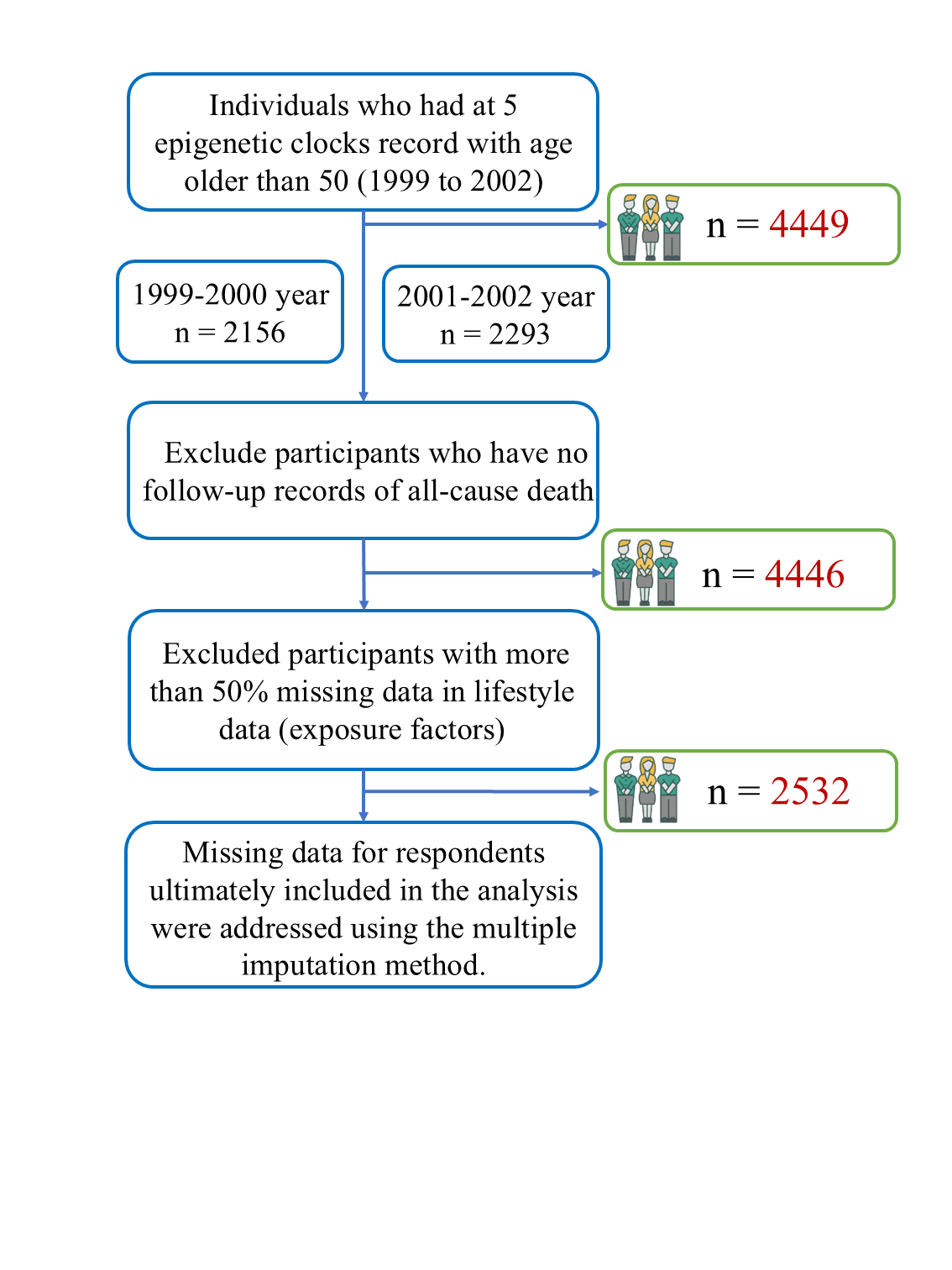
**

**eFigure 1**. Flow diagram detailing the inclusion and exclusion criteria for participant selection in the study.

**
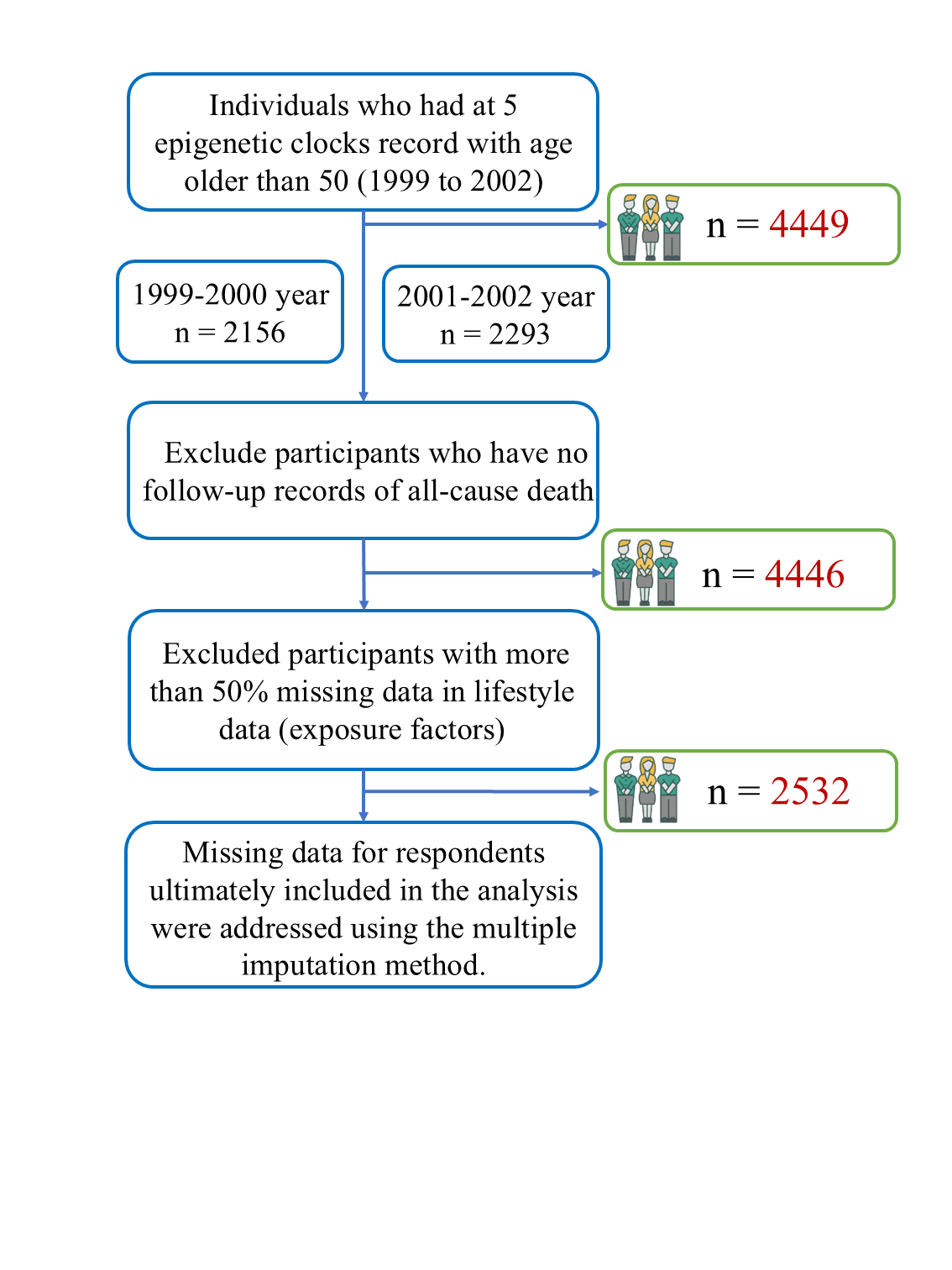
**

**eFigure 2.** The area under the curves (AUC) of the five epigenetic age acceleration models using time-dependent receiver operating characteristic (ROC) curve without adjusted sex and age.

**
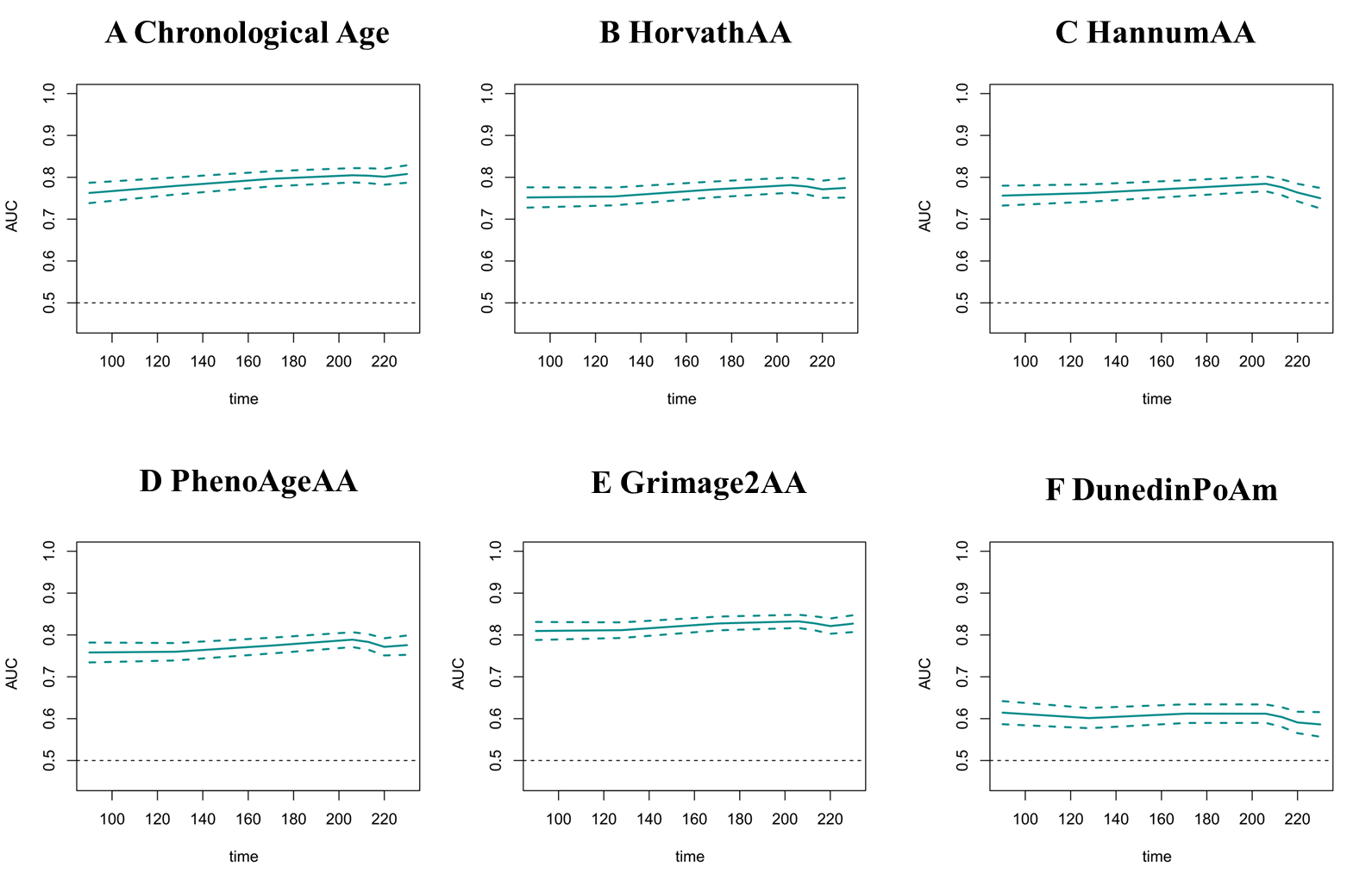
**

**eFigure 3.** Propensity score matching was applied to adjust for the effects of age and sex in evaluating the clinical significance of epigenetic age acceleration.


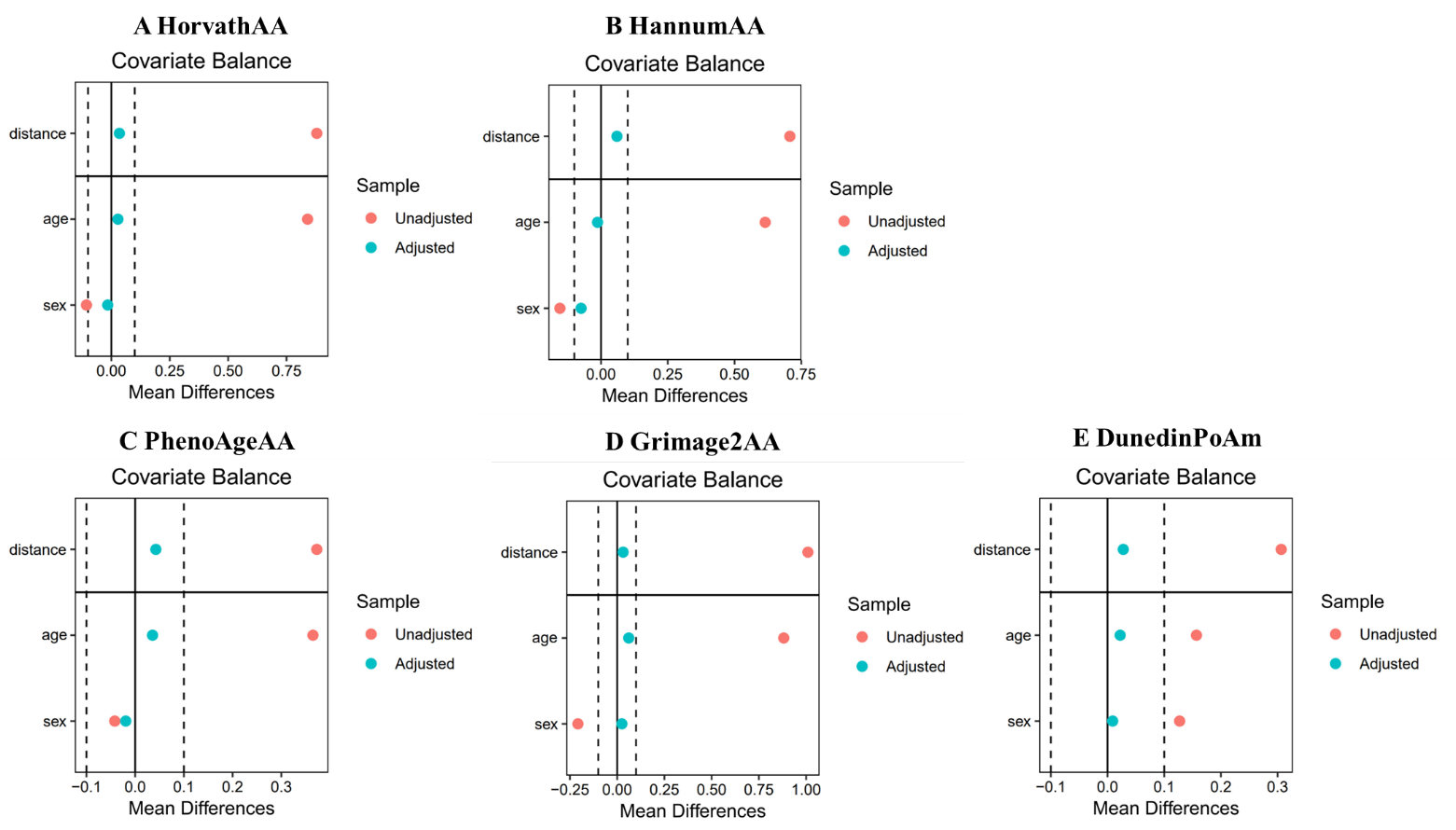


**eFigure 4**. Subgroup analyses for the association between the healthy lifestyle and PhenoAgeAA by sex, age, hypertension, diabetes, cancer and CVD. Models adjusted for age, sex, ethnicity, marital status, educational level, household income, hypertension, diabetes, cardiovascular disease), and cancer.


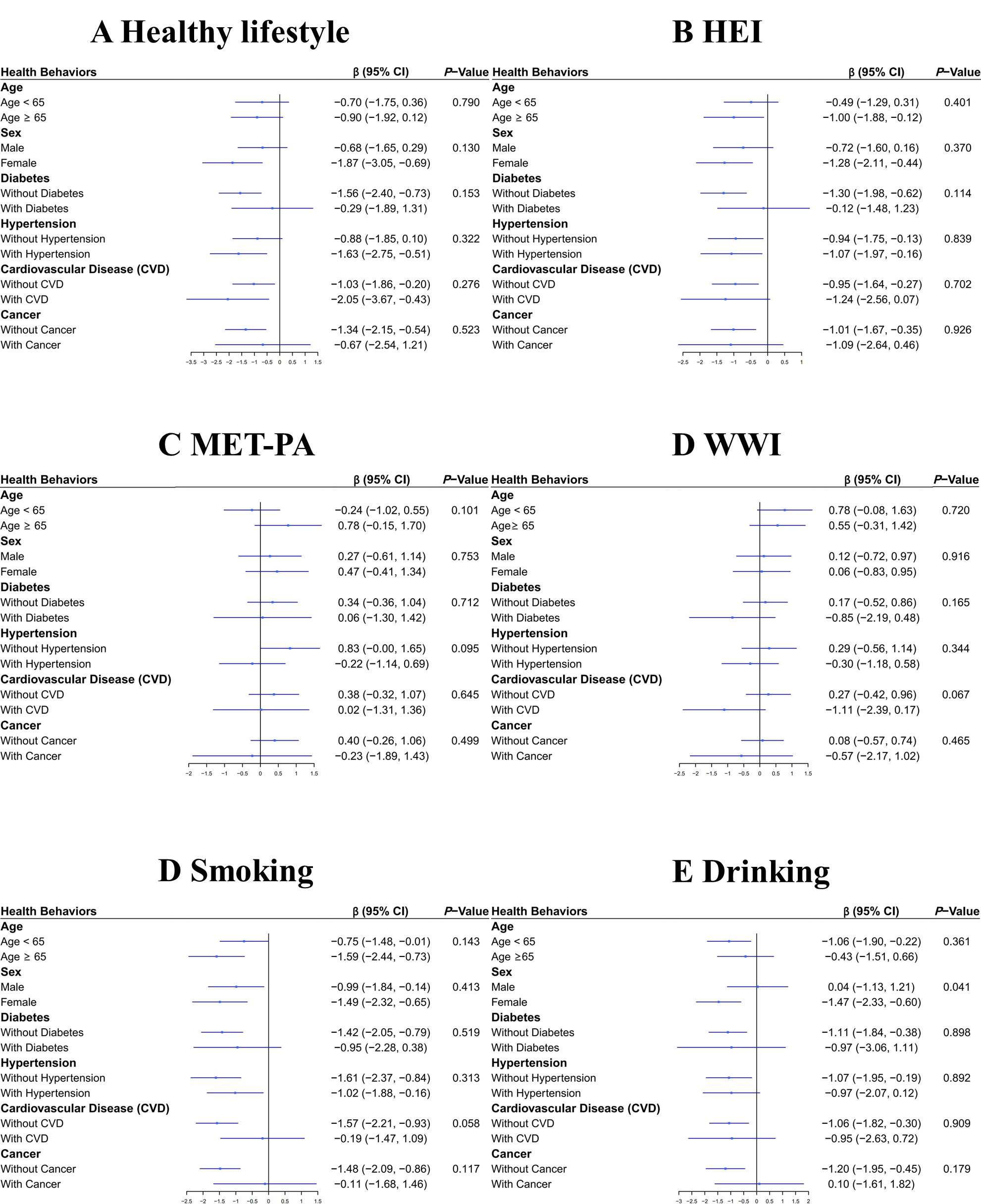


**eFigure 5.** Subgroup analyses for the association between the healthy lifestyle and GrimAge2AA by sex, age, hypertension, diabetes, cancer and CVD. Models adjusted for age, sex, ethnicity, marital status, educational level, household income, hypertension, diabetes, cardiovascular disease), and cancer.


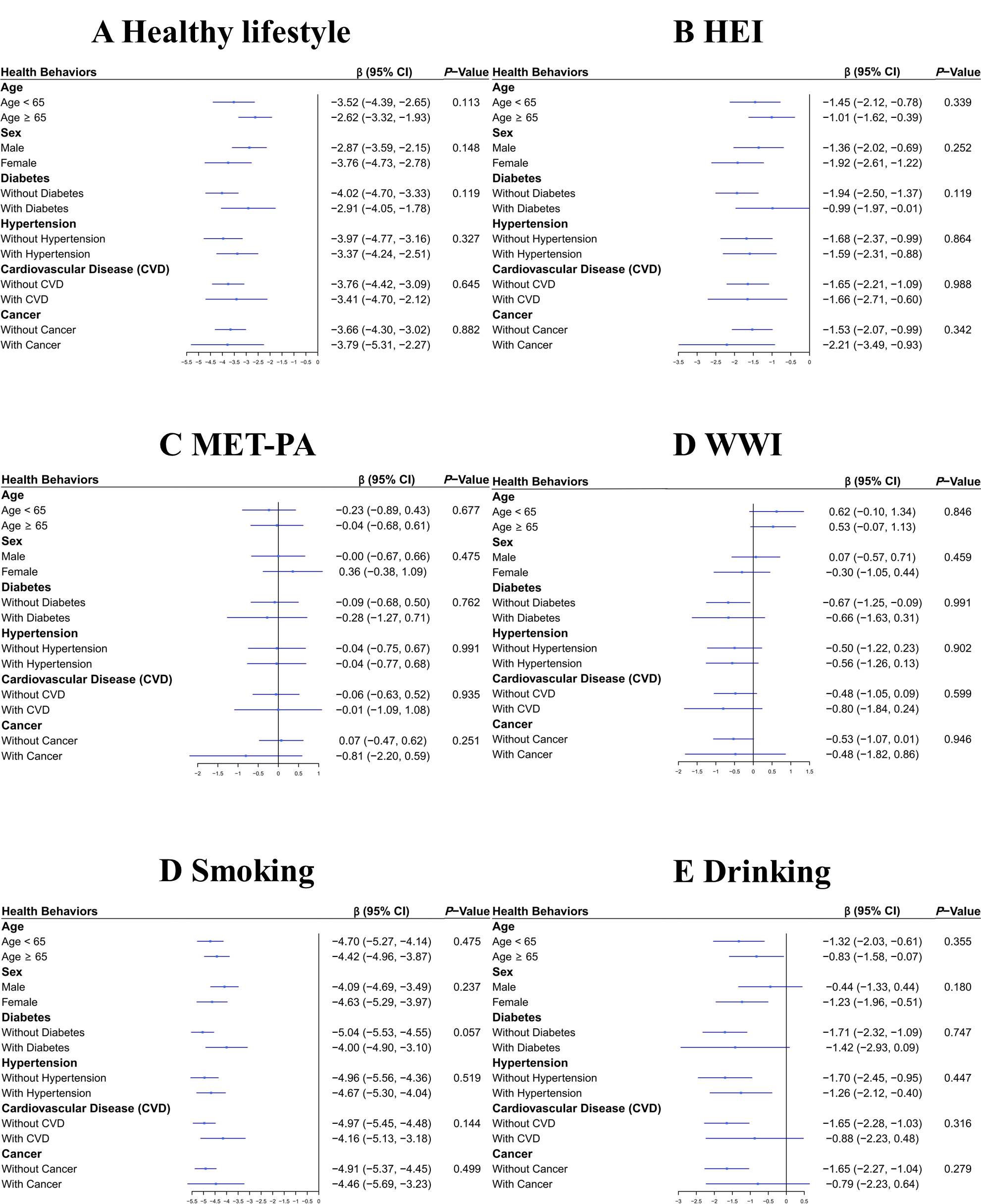


**eFigure 6.** Subgroup analyses for the association between the healthy lifestyle and DunedinPoAm by sex, age, hypertension, diabetes, cancer and CVD. Models adjusted for age, sex, ethnicity, marital status, educational level, household income, hypertension, diabetes, cardiovascular disease), and cancer.


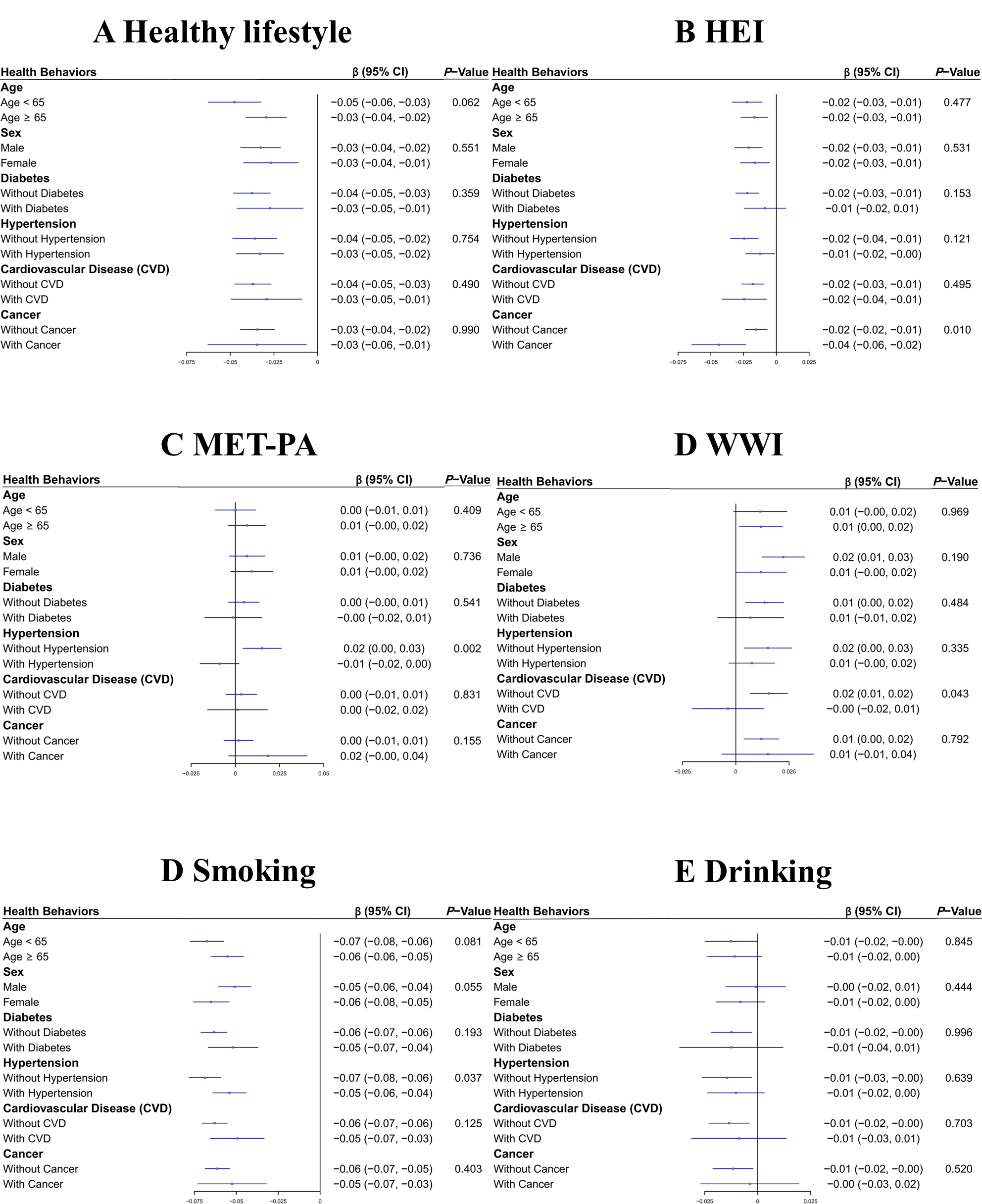


**eTable 1.** **PhenoAgeAA，GrimAge2AA and DunedinPoAm sensitivity analysis Model 1-6.**

| All cause | **Model 1** | |  | **Model 2** | |  | **Model 3** | |  | **Model 4** | |  | **Model 5** | |  | **Model 6** | |
| --- | --- | --- | --- | --- | --- | --- | --- | --- | --- | --- | --- | --- | --- | --- | --- | --- | --- |
|  | β(95%CI) | *P* value |  | β(95%CI) | *P* value |  | β(95%CI) | *P* value |  | β(95%CI) | *P*  value |  | β(95%CI) | *P*  value |  | β(95%CI) | *P*  value |
| PhenoAgeAA | -1.33  (-2.06 to-0.60) | <.001 |  | -1.11  (-1.95 to-0.27) | 0.01 |  | -1.40  (-2.16 to-0.65) | <.001 |  | -1.41  (-2.03 to-0.80) | <.001 |  | -1.28  (-2.47 to-0.09) | 0.03 |  | -2.11  (-3.45 to-0.76) | <.001 |
| GrimAge2AA | -3.89  (-4.46 to-3.31) | <.001 |  | -3.87  (-4.53 to-3.21) | <.001 |  | -3.84  (-4.43 to-3.24) | <.001 |  | -3.60  (-4.14 to-3.06) | <.001 |  | -4.13  (-5.12 to-3.15) | <.001 |  | -4.55  (-5.53 to-3.57) | <.001 |
| DunedinPoAm | -0.03  (-0.04 to-0.03) | <.001 |  | -0.03  (-0.05 to-0.02) | <.001 |  | -0.04  (-0.04 to-0.03) | <.001 |  | -0.03  (-0.04 to-0.02) | <.001 |  | -0.03  (-0.05 to-0.02) | <.001 |  | -0.04  (-0.05 to-0.02) | <.001 |

Model 1 was evaluated the association between the total score of the original healthy lifestyle and EAA.

Model 2 was evaluated the association of healthy lifestyle scores with EAA in participants who only including complete data.

Model 3 was evaluated the association of healthy lifestyle scores with EAA. in participants who exclude extreme values of lifestyle adherence scores (0 point or 10 point).

Model 4 was evaluated the association of healthy lifestyle scores with EAA in participants who excluded extreme methylation age accelerations (Data points that fall below Q1 − 1.5IQR or above Q3 + 1.5IQR are considered potential outliers).

Model 5 was evaluated the association of healthy lifestyle scores with EAA in participants who excluded self-reported CVD, cancer, or diabetes.

Model 6 was evaluated the association of healthy lifestyle scores with EAA conducting the analysis using propensity score matching based on the same covariates applied in the primary models.

Multivariate linear regression analysis adjusted demographic variables (age, sex, ethnicity, education level, marital and annual household income) and health status (hypertension, diabetes, cancer, heart diseases).

EAA: epigenetic age acceleration
